# Prevalence and Mortality of Sarcopenia in a Community-dwelling Older Japanese Population: The Hisayama Study

**DOI:** 10.2188/jea.JE20190289

**Published:** 2021-05-05

**Authors:** Kimitaka Nakamura, Daigo Yoshida, Takanori Honda, Jun Hata, Mao Shibata, Yoichiro Hirakawa, Yoshihiko Furuta, Hiro Kishimoto, Tomoyuki Ohara, Takanari Kitazono, Yasuharu Nakashima, Toshiharu Ninomiya

**Affiliations:** 1 Department of Epidemiology and Public Health, Graduate School of Medical Sciences, Kyushu University, Fukuoka, Japan; 2 Department of Orthopaedic Surgery, Graduate School of Medical Sciences, Kyushu University, Fukuoka, Japan; 3 Center for Cohort Studies, Graduate School of Medical Sciences, Kyushu University, Fukuoka, Japan; 4 Department of Medicine and Clinical Science, Graduate School of Medical Sciences, Kyushu University, Fukuoka, Japan; 5 Faculty of Arts and Science, Kyushu University, Fukuoka, Japan; 6 Department of Neuropsychiatry, Graduate School of Medical Sciences, Kyushu University, Fukuoka, Japan

**Keywords:** sarcopenia, Asian Working Group for Sarcopenia, prevalence, mortality

## Abstract

**Background:**

The prevalence of sarcopenia defined using the Asian Working Group for Sarcopenia (AWGS) criteria in Asian communities has not been fully addressed. Moreover, few studies have addressed the influence of sarcopenia on mortality.

**Methods:**

A total of 1,371 and 1,597 residents aged 65 years or older participated in health surveys in 2012 and 2017. Sarcopenia was determined using the AWGS definition. Factors associated with the presence of sarcopenia were assessed using a logistic regression model in participants in the 2012 survey. Subjects in the 2012 survey were followed-up prospectively for a median of 4.3 years. Mortality risk for subjects with sarcopenia was examined using the Cox proportional hazards model.

**Results:**

The crude prevalence of sarcopenia was 7.4% and 6.6% in participants at the 2012 and 2017 surveys, respectively; there was no significant difference between surveys (*P* = 0.44). The prevalence of sarcopenia increased significantly with age in both sexes (both *P* for trend <0.001). Subjects with sarcopenia were more likely to exercise less regularly, to intake less total energy, and to exhibit a disability in activity of daily living than those without. The multivariable-adjusted hazard ratio for all-cause mortality was 2.20 (95% confidence interval, 1.25–3.85) in subjects with sarcopenia, compared to those without.

**Conclusions:**

Approximately 7% of older subjects had sarcopenia in a community-dwelling older Japanese population. Moreover, subjects with sarcopenia had an increased mortality risk. Our findings suggest that a public health strategy for sarcopenia is needed to extend healthy life expectancy.

## INTRODUCTION

Sarcopenia is a multi-faceted geriatric syndrome characterized by the age-related decline of skeletal muscle mass plus low muscle strength and/or physical performance. Subjects with sarcopenia have been reported to be at increased risk of the development of adverse health outcomes such as morbidity, disability, and mortality.^1^^,^^2^ Asian countries constitute a rapidly aging region with a huge population.^1^ Therefore, sarcopenia and its effects on the health of older people have become a significant public health problem in Asian countries.

Despite the increasing significance of sarcopenia, there was no consensual definition of sarcopenia until a decade ago.^2^ In 2010, the European Working Group on Sarcopenia in Older People (EWGSOP) proposed a diagnostic definition of sarcopenia based on skeletal muscle mass measurement, muscle strength measured by handgrip, and physical performance measured by gait speed, and their definition has become the most widely used worldwide.^3^ In 2014, the Asian Working Group for Sarcopenia (AWGS) proposed modified cutoff values to render the measurements used in the EWGSOP definition for Asian populations.^1^ Several studies have reported that the prevalence of sarcopenia in Asian countries is 7–10%.^4^^–^^7^ Additionally, epidemiological evidence revealed that sarcopenia defined using the EWGSOP criteria was significantly associated with greater risks of adverse health outcomes, such as mortality, activities of daily living (ADL) decline, falls, fractures, and hospitalization.^8^ However, in most of these studies, the subjects were volunteers, and there have been few community-based studies addressing these issues.^4^^,^^9^

The aim of the current study was to examine the prevalence of sarcopenia defined using the AWGS criteria using a cross-sectional community-based sample of a Japanese older population. We also investigated the influence of sarcopenia on mortality risk based on the data from a prospective longitudinal study conducted in a community-dwelling older Japanese population.

## METHODS

### Study population

The Hisayama Study was established in 1961 in the town of Hisayama, a suburb of the metropolitan area of Kyushu Island in Japan.^10^ Since 1961, the age and occupational distributions and the nutrient intake of residents in this town have been similar to those of Japan as a whole based on data from the national census and nutrition survey.^11^ Full community surveys of the residents aged 40 years or older have been repeated annually.^10^ Additionally, comprehensive screening surveys of cognitive function and ADL for older residents have been repeated every 6–7 years since 1985.^12^ In 2012, a total of 1,906 residents aged 65 years or older (participation rate, 93.6%) participated in the screening survey. After excluding 44 subjects who did not consent to participate in the present study and 491 subjects without sufficient information to determine a diagnosis of sarcopenia, the remaining 1,371 subjects (601 men and 770 women) were enrolled in the current study (final participation rate, 67.3%). Similarly, a total of 1,597 (706 men and 891 women) of the 2,202 residents aged 65 years or older who participated in the survey in 2017 (participation rate, 94.1%) were included after excluding 102 subjects who did not consent to participate in the present study and 503 subjects for whom there was insufficient information to determine a diagnosis of sarcopenia (final participation rate, 68.2%). A detailed flow diagram of the subject-selection process is shown in eFigure 1.

The current study was conducted with the approval of the Kyushu University Institutional Review Board for Clinical Research, and written informed consent was obtained from all participants.

### Definition of sarcopenia

Sarcopenia was defined as the presence of both low muscle mass and low muscle function (either low handgrip strength or low gait speed) according to the AWGS definition.^1^ For subjects who were missing data for either muscle mass or muscle function, we attempted to determine whether sarcopenia was present using the limited data available. The detailed diagnostic patterns of sarcopenia for the 2012 and 2017 surveys are shown in eTable 1. Muscle mass was measured by bioelectrical impedance analysis using an MC-190 body composition analyzer (Tanita, Tokyo, Japan). Appendicular skeletal muscle mass (ASM) was calculated as the sum of skeletal muscle mass in the arms and legs. Absolute ASM was converted to the skeletal muscle mass index (SMI) by dividing by height in meters squared (kg/m^2^).^13^ Low muscle mass was defined as SMI of <7.0 kg/m^2^ for men and <5.7 kg/m^2^ for women.^1^ Handgrip strength was measured using a digital strength dynamometer (T.K.K.5401; Takei Scientific Instruments) according to the instructions provided by trained personnel or nurse. The participants were encouraged to exert maximal handgrip strength. Two trials were recorded alternately for each hand, and the maximum value among the four measurements was used. Low handgrip strength was defined as <26 kg for men and <18 kg for women.^1^ Usual gait speed was not measured at the survey in 2012 because this survey was conducted before the AWGS definition was made available. Therefore, the maximum gait speed was used instead of the usual gait speed. Maximum gait speed was tested twice in the middle 5 meters of the course and the faster of the two measurements was used for the analysis. The cut-off values for maximum gait speed corresponding to those for usual gait speed in the AWGS definition (0.8 m/s for both men and women) were calculated by using sex-specific linear regression equations among 1,455 Hisayama residents aged 65 years or older, whose usual gait speed and maximum gait speed data were collected in 2017 (eFigure 2). In this survey, low gait speed was defined as a maximum gait speed <1.25 m/s for men and <1.15 m/s for women.

### Other risk factors

A self-administered questionnaire concerning smoking habits, alcohol intake, physical activity, living arrangement, antihypertensive agent use, use of insulin and oral glucose-lowering agents, history of cardiovascular disease or cancer, and history of fracture was completed by each subject and was checked by trained interviewers at the surveys in 2012 and 2017. Smoking habits and alcohol intake were categorized as current use or not. Regular exercise was defined as engaging in exercise ≥3 times per week during leisure time. ADL disability was defined as the Barthel Index of ≤95.^14^^–^^17^ Cognitive impairment was defined as mild cognitive impairment or dementia according to the criteria of Peterson and the Diagnostic and Statistical Manual of Mental Disorders, Third Edition, Revised through a screening survey using the Mini-Mental State Examination and comprehensive investigations by psychiatrists as previously described.^12^^,^^18^ A dietary survey was conducted using a Semi-Quantitative Food Frequency Questionnaire concerning food intake.^19^ Blood pressure was measured three times using an automated sphygmomanometer with the subject seated after at least 5 min rest and the mean of the three measurements was calculated. Hypertension was defined as blood pressure ≥140/90 mm Hg or current use of antihypertensive agents.^20^ Obesity and leanness were defined as body mass index (BMI) ≥25.0 kg/m^2^ and <18.5 kg/m^2^, respectively. Blood samples were collected from an antecubital vein after an overnight fast. Plasma glucose levels were measured using the hexokinase method. Diabetes mellitus was defined as fasting plasma glucose levels ≥7.0 mmol/L, 2-hr post load or casual glucose levels ≥11.1 mmol/L, or current use of oral glucose-lowering agents or insulin.^21^ The total cholesterol level was measured enzymatically. Hypercholesterolemia was defined as serum total cholesterol levels ≥5.69 mmol/L or current use of lipid-lowering medication. Serum albumin was analyzed with the bromocresol green method.

### Follow-up survey

The participants at the survey in 2012 were followed prospectively from the date of a comprehensive assessment to November 2016. As described in a previous report,^11^ their health status was checked yearly via mail or telephone for any subjects who did not undergo the annual examination, or who moved from the town. We also established a daily monitoring system among the study team, local physicians, and members of the town’s health and welfare office to receive information on any deaths. All subjects were completely followed up.

### Statistical analysis

Differences in the mean values and frequencies of risk factors between surveys or between subjects with and without sarcopenia were tested using the *t* test and χ^2^ test, respectively. 95% confidence intervals (CIs) for the prevalence of sarcopenia were estimated according to the binomial distribution. Trends in the prevalence of sarcopenia between surveys or age groups were tested using logistic regression analysis. Odds ratios with 95% CIs of the associated factors for the presence of sarcopenia were estimated using logistic regression analysis. The survival rates for participants with and without sarcopenia were computed using the Kaplan-Meier method and were compared using a log-rank test, where participants were censored at the date of their death or the end of follow-up. The Cox proportional hazards model was used to estimate the hazard ratios (HRs) with 95% CIs for all-cause deaths. As a sensitivity analysis, we repeated the analysis after excluding subjects who died during the first 2 years of the follow-up period. The heterogeneity in the relationship between subgroups was tested by adding interaction terms to the relevant Cox model. The software package SAS (version 9.4; SAS Institute, Cary, NC, USA) was used to perform all statistical analyses. Two-sided values of *P* < 0.05 were considered statistically significant in all analyses.

## RESULTS

**Figure 1.**
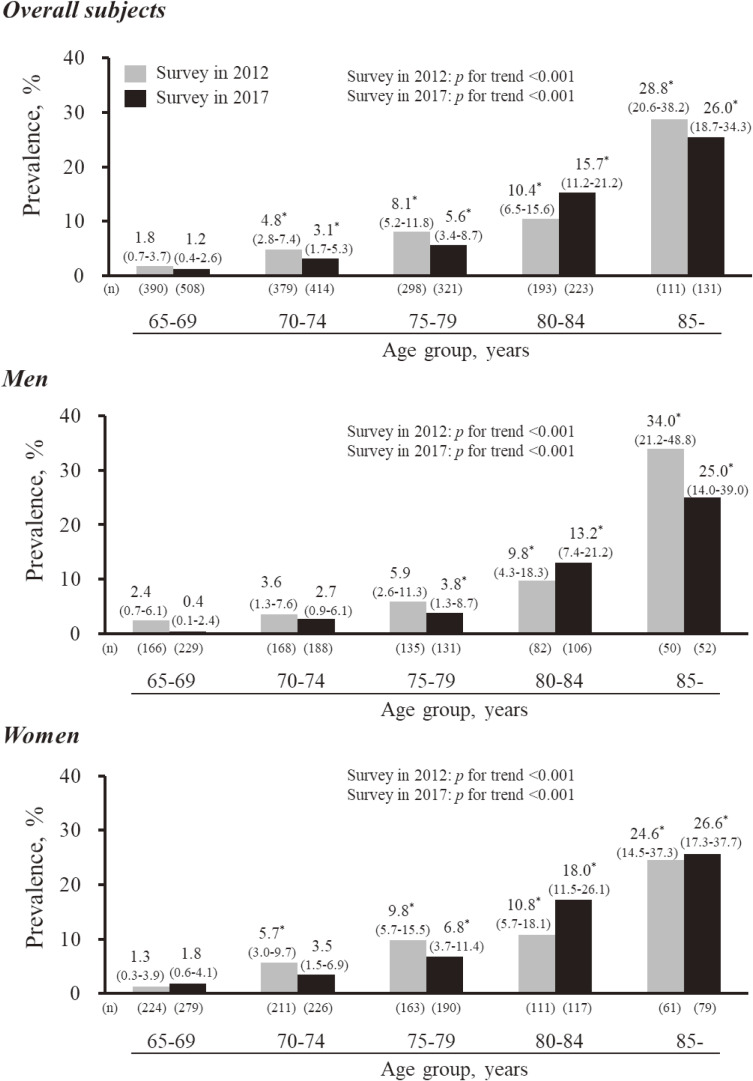
Age-specific prevalence of sarcopenia in men, women and subjects overall at the 2012 and 2017 surveys. Values on each bar are shown as the age-specific prevalence (95% confidence interval). The maximum gait speed was used for estimating sarcopenia. ^*^P < 0.05 vs 65–69 years in each survey. There was no evidence of a significant difference in the age-specific prevalence of sarcopenia between surveys for all age categories in men, women, or subjects overall (all *P* > 0.12).

The characteristics of the study participants in the surveys in 2012 and 2017 are shown in Table 1. The mean ages among the study participants in 2012 and 2017 were 74.2 and 74.1 years, respectively. The mean values of serum albumin, total energy intake, and protein- and fat-energy ratio and the frequencies of living alone and hypercholesterolemia were significantly higher in participants at the survey in 2017 than those at the survey in 2012. Regarding the components of sarcopenia, there were no differences in the mean values of SMI, handgrip strength, maximum gait speed, or the frequencies of low muscle mass, low handgrip strength or low gait speed, between the surveys. Similar findings were observed in either sex, except for living alone and maximum gait speed in men and low gait speed in women. The crude prevalence of sarcopenia in 2012 and 2017 was 7.4% (95% CI, 6.0–8.9) and 6.6 (95% CI, 5.5–8.0), respectively, for the overall population, and there was no significant difference between surveys (*P* = 0.44) (Table 2). The trend in the sex-specific prevalence of sarcopenia tended to decline marginally between surveys in men (7.2% and 5.4%, *P* = 0.19), but remained on a plateau in women (7.5% and 7.6%, *P* = 0.94). The prevalence of sarcopenia increased significantly with age for overall subjects and both sexes (both *P* for trend <0.001) at the 2012 and 2017 surveys (Figure 1). There was no significant difference in the age-specific prevalence of sarcopenia between surveys for all age categories in either men, women or overall subjects (all *P* > 0.12). The sensitivity analysis using the usual gait speed for estimating sarcopenia in 2017 found that the prevalence of sarcopenia was 6.3% (95% CI, 5.2–7.6) for overall subjects (eTable 2).

**Table 1.** Clinical characteristics of participants in the 2012 and 2017 surveys

Variables	Overall subjects		Men		Women	
		
	Survey in 2012	Survey in 2017	Survey in 2012	Survey in 2017	Survey in 2012	Survey in 2017
	(*n* = 1,371)	(*n* = 1,597)	(*n* = 601)	(*n* = 706)	(*n* = 770)	(*n* = 891)
Age, years	74.2 (6.5)	74.1 (6.7)	74.1 (6.3)	73.9 (6.6)	74.3 (6.6)	74.2 (6.7)
Sex, women, %	56.2	55.8	0	0	100	100
Height, cm	154.2 (8.9)	155.5 (9.3)^**^	161.6 (6.1)	163.3 (6.5)^**^	148.4 (6.0)	149.4 (6.1)^**^
Weight, kg	55.4 (10.1)	56.6 (10.7)^**^	60.9 (9.1)	62.7 (9.4)^**^	51.0 (8.5)	51.7 (9.0)
BMI, kg/m^2^	23.2 (3.3)	23.3 (3.4)	23.3 (3.0)	23.5 (3.0)	23.1 (3.5)	23.2 (3.7)
Obesity, %	26.1	29.2	25.0	28.5	27.0	29.7
Leanness, %	6.4	6.6	5.2	3.9	7.4	8.7
Living alone, %	10.0	12.9^*^	5.2	7.1	13.7	17.5^*^
Hypertension, %	70.8	68.1	71.8	70.3	70.0	66.3
Diabetes, %	24.6	26.1	30.9	33.3	19.7	20.4
Hypercholesterolemia, %	55.3	63.6^**^	40.8	48.8^**^	66.7	75.3^**^
Serum albumin, g/dL	4.1 (0.2)	4.2 (0.3)^**^	4.1 (0.3)	4.2 (0.3)^**^	4.1 (0.2)	4.3 (0.3)^**^
History of CVD or cancer, %	27.4	27.2	36.9	33.4	20.0	22.3
Cognitive impairment, %	20.1	17.8	21.5	18.3	19.0	17.4
History of fracture, %	36.7	n.a.	36.1	n.a.	37.1	n.a.
ADL disability, %	5.8	7.6	5.0	5.8	6.4	9.0
Smoking habits, %	8.3	8.3	16.0	14.8	2.2	3.2
Alcohol intake, %	41.1	43.4	63.2	66.7	23.9	25.0
Regular exercise, %	18.9	21.3	21.2	24.4	17.1	18.8
Total energy intake, kcal/day	1,531 (338)	1,733 (387)^**^	1,669 (358)	1,875 (399)^**^	1,423 (276)	1,620 (336)^**^
Energy balance, % of total energy intake						
Protein, %	12.4 (1.9)	14.3 (2.3)^**^	12.0 (1.9)	13.5 (2.2)^**^	12.8 (1.9)	15.0 (2.0)^**^
Fat, %	25.3 (5.1)	31.0 (5.7)^**^	24.2 (4.8)	28.9 (5.6)^**^	26.2 (5.1)	32.7 (5.3)^**^
Carbohydrate, %	57.1 (7.2)	49.4 (7.2)^**^	55.1 (8.0)	48.6 (8.2)^**^	58.6 (6.2)	50.1 (6.3)^**^
SMI, kg/m^2^	6.9 (1.1)	6.9 (1.1)	7.6 (1.0)	7.7 (1.0)	6.2 (0.7)	6.2 (0.7)
Low muscle mass, %	22.8	21.7	23.3	19.4	22.3	23.5
Handgrip strength, kg	27.3 (8.3)	27.6 (8.7)	34.3 (6.7)	34.9 (7.0)	21.8 (4.3)	21.8 (4.5)
Low handgrip strength, %	14.7	15.2	10.2	9.8	18.2	19.4
Maximum gait speed, m/s	1.74 (0.41)	1.72 (0.33)	1.85 (0.41)	1.76 (0.35)^**^	1.65 (0.39)	1.68 (0.31)
Usual gait speed, m/s	n.a.	1.28 (0.25)	n.a.	1.27 (0.25)	n.a.	1.29 (0.25)
Low gait speed, %^a^	7.1	5.3	5.8	5.7	8.1	5.0^*^

ADL, activities of daily living; BMI, body mass index; CVD, cardiovascular disease; n.a., not available; SMI, skeletal muscle mass index.

Values are shown as the means (standard deviations) or frequencies.

^a^The maximum gait speed was used for estimating low gait speed because the data on usual gait speed were unavailable in the 2012 survey.

^*^P < 0.05, ^**^P < 0.01 vs survey in 2012.

**Table 2.** Crude prevalence of sarcopenia in participants at the 2012 and 2017 surveys

	Participants at the survey in 2012	Participants at the survey in 2017	*P* value
***Overall subjects***			
Subjects with sarcopenia/total subjects	101/1,371	106/1,597	
Crude prevalence, %^a^	7.4 (6.0–8.9)	6.6 (5.5–8.0)	0.44

***Men***			
Subjects with sarcopenia/total subjects	43/601	38/706	
Crude prevalence, %^a^	7.2 (5.2–9.5)	5.4 (3.8–7.3)	0.19

***Women***			
Subjects with sarcopenia/total subjects	58/770	68/891	
Crude prevalence, %^a^	7.5 (5.8–9.6)	7.6 (6.0–9.6)	0.94

Values are shown as the crude prevalence (95% confidence interval).

^a^The maximum gait speed was used for estimating sarcopenia because the data on usual gait speed were unavailable in the 2012 survey.

We also investigated the factors associated with the presence of sarcopenia (Table 3). Subjects with older age and ADL disability were significantly more likely to have sarcopenia, whereas subjects with regular exercise and higher total energy intake were significantly less likely to have sarcopenia. These associations were not altered substantially in the multivariable-adjusted analysis.

**Table 3.** Factors associated with the presence of sarcopenia among participants at the 2012 survey

Variables	Age- and sex-adjusted		Fully adjusted	
	
	Odds ratio (95% CI) on the presence of sarcopenia^a^	*P* value	Odds ratio (95% CI) on the presence of sarcopenia^a^	*P* value
Age, per 1 year	1.12 (1.11–1.18)^b^	<0.001	1.12 (1.08–1.16)	<0.001
Women, vs men	1.01 (0.66–1.55)^b^	0.96	0.87 (0.51–1.49)	0.62
Living alone, vs live with someone	1.21 (0.61–2.41)	0.58	1.12 (0.55–2.28)	0.76
Hypertension, yes vs no	0.68 (0.42–1.09)	0.11	0.69 (0.41–1.15)	0.16
Diabetes, yes vs no	0.89 (0.53–1.48)	0.64	0.90 (0.53–1.55)	0.71
Hypercholesterolemia, yes vs no	0.79 (0.51–1.23)	0.30	0.86 (0.54–1.37)	0.51
Serum albumin, per 0.1 g/dL	0.93 (0.86–1.02)	0.11	0.96 (0.88–1.05)	0.40
History of CVD or cancer, yes vs no	1.30 (0.83–2.05)	0.26	1.26 (0.78–2.03)	0.35
Cognitive impairment, yes vs no	1.47 (0.93–2.33)	0.10	1.24 (0.75–2.02)	0.40
History of fracture, yes vs no	1.07 (0.69–1.64)	0.78	1.11 (0.71–1.74)	0.66
ADL disability, yes vs no	2.89 (1.60–5.24)	<0.001	2.56 (1.35–4.85)	0.004
Smoking habits, yes vs no	1.63 (0.72–3.66)	0.24	1.54 (0.65–3.61)	0.32
Alcohol intake, yes vs no	0.77 (0.47–1.28)	0.32	0.96 (0.56–1.62)	0.87
Regular exercise, yes vs no	0.41 (0.19–0.87)	0.02	0.41 (0.19–0.89)	0.02
Total energy intake, per 100 kcal/day	0.86 (0.79–0.93)	<0.001	0.88 (0.81–0.95)	0.002

ADL, activities of daily living; CI, confidence interval: CVD, cardiovascular disease.

^a^The maximum gait speed was used for estimating sarcopenia because the data on usual gait speed were unavailable in the 2012 survey.

^b^Age is sex-adjusted and sex is age-adjusted.

**Figure 2.**
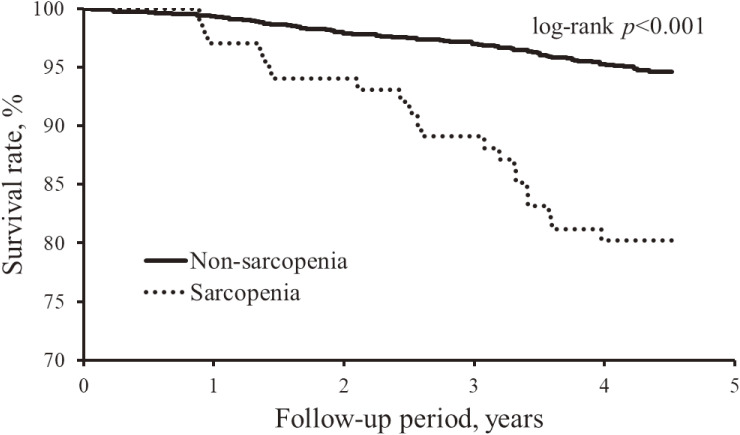
Crude cumulative survival rate according to the presence of sarcopenia. The maximum gait speed was used for estimating sarcopenia.

Finally, we investigated the influence of sarcopenia on mortality. The baseline characteristics of participants at the survey in 2012 according to the status of sarcopenia are shown in eTable 3. During the follow-up period (median, 4.3 years; interquartile range, 4.3–4.4 years), a total of 87 subjects died. In the Kaplan-Meier analysis, there was a significant difference in the crude survival rate between subjects with and without sarcopenia (log-rank *P* < 0.001; Figure 2). Subjects with sarcopenia had a 2.2-fold higher risk of all-cause mortality than those without after adjusting for confounding factors (Table 4).

**Table 4.** Risk of all-cause mortality in subjects with sarcopenia or its component than those without (2012–2016)

	Number of events/subjects	Mortality rate (per 1,000 PYs)^b^	Age- and sex-adjusted		Multivariable-adjusted^c^	
	
			Hazard ratio (95% CI)	*P* value	Hazard ratio (95% CI)	*P* value
***Sarcopenia^a^***						
Absence	67/1,270	12.4	1.00 (reference)		1.00 (reference)	
Presence	20/101	50.0	2.46 (1.43–4.22)	0.001	2.20 (1.25–3.85)	0.006
***Components of the definition for Sarcopenia***						
***Low muscle mass***						
Absence	56/1,005	13.2	1.00 (reference)		1.00 (reference)	
Presence	26/296	21.2	1.13 (0.69–1.82)	0.63	1.10 (0.67–1.80)	0.72
***Low handgrip strength***						
Absence	55/1,170	11.0	1.00 (reference)		1.00 (reference)	
Presence	32/201	39.8	2.80 (1.74–4.51)	<0.001	2.56 (1.55–4.24)	<0.001
***Low gait speed^a^***						
Absence	60/1,153	12.2	1.00 (reference)		1.00 (reference)	
Presence	15/88	43.0	2.44 (1.34–4.44)	0.004	2.02 (1.07–3.83)	0.03

ADL, activities of daily living; CI, confidence interval; PYs, person-years.

^a^The maximum gait speed was used for estimating sarcopenia and low gait speed because the data on usual gait speed were unavailable in the 2012 survey.

^b^The mortality rates were calculated using the person-year method. Values were unadjusted.

^c^Adjusted for age, sex, hypertension, diabetes, history of cardiovascular disease or cancer, history of fracture, ADL disability, smoking habits, alcohol intake, regular exercise, and total energy intake. Subjects with missing covariates data (*n* = 56) were excluded from the multivariable-adjusted analyses.

Regarding the components of the definition for sarcopenia, low handgrip strength or low gait speed were associated with a significant, approximately 2-fold higher risk of all-cause mortality, whereas no clear association was detected between muscle mass and mortality. Moreover, we also investigated the associations of sarcopenia or its components with all-cause mortality after adjusting for either SMI, handgrip strength, or maximum gait speed in addition to the traditional risk factors (eTable 4). As a result, the magnitude of the excess mortality risk of sarcopenia was attenuated after adjusting for handgrip strength or maximum gait speed, but not after adjusting for SMI.

Regarding the components for the definition of sarcopenia, low handgrip strength was significantly associated with mortality risk after additional adjustment for either SMI or maximum gait speed. In the analysis with continuous variables, on the other hand, the mortality risk increased with decreasing maximum gait speed after additional adjusting for either SMI or handgrip strength. In the subgroup analyses, there was no evidence of heterogeneity in the influence of sarcopenia on the risk of all-cause death between the subgroups of risk factors (eTable 5).

## DISCUSSION

The current study demonstrated that the prevalence of sarcopenia diagnosed using the AWGS criteria was 7.4% in 2012 and 6.6% in 2017 in a community-dwelling older Japanese population. The prevalence of sarcopenia showed a declining trend in men, but remained on a plateau in women. The prevalence of sarcopenia increased significantly with age. Older subjects and subjects with ADL disability, less regular exercise, and less total energy intake were more likely to have sarcopenia. Additionally, subjects with sarcopenia were at significantly higher risk of all-cause mortality than those without sarcopenia after adjusting for potential confounding factors. Regarding the components of the definition of sarcopenia, low handgrip strength and low gait speed contributed to the excess mortality risk.

A recent systematic review revealed that the estimated prevalence of sarcopenia was 10% for both sexes among adults aged ≥60 years, but the included studies used three different sets of diagnostic criteria to diagnose sarcopenia (ie, the EWGSOP, International Working Group on Sarcopenia, and AWGS definitions).^22^ Although several studies have reported the prevalence of sarcopenia defined using the AWGS criteria, most of these were conducted on volunteers, such as participants in physical examination programs.^4^^–^^6^ Therefore, it would be valuable to investigate the prevalence of sarcopenia based on the AWGS definition in Asian communities. One community-based study of Chinese elderly reported that the prevalence of sarcopenia defined using the AWGS criteria was 9.8%.^7^ This finding is close to ours, but the results may not be directly comparable due to the different methods used to measure muscle mass (calf circumference vs bioelectrical impedance analysis) and the different participation rates (46% vs >67%) between the studies. Further community-based studies on sarcopenia prevalence in Asian populations are needed to clarify the burden of sarcopenia in the Asian community.

In the present study, the prevalence of sarcopenia increased significantly with older age for both sexes among the participants at the survey in 2012 and 2017, which agreed with the previous reports.^6^^,^^23^^–^^27^ Moreover, we identified a decreasing trend in the prevalence of sarcopenia between the surveys in men. This tendency may reflect the play of chance, but it may also be related to the improvement in nutrition status (eg, the mean values of total energy intake, protein-energy ratio, and serum albumin were higher at the 2017 than the 2012 survey). However, more surveys will be needed to confirm this decreasing trend, since it was based on data from only two surveys.

In the present study, subjects with regular exercise and higher total energy intake had a significantly lower likelihood of sarcopenia, whereas ADL disability was associated with a significantly higher likelihood of sarcopenia. Several observational studies demonstrated that lower physical activity and/or undernutrition was significantly associated with the higher prevalence of sarcopenia.^28^^–^^30^ Additionally, a systematic review of intervention studies demonstrated that exercise and nutrition interventions were effective for reducing the risk of developing sarcopenia.^31^ The present study also revealed that there was a significant positive association between sarcopenia and ADL disability in the cross-sectional analysis, probably suggesting that sarcopenia induced ADL disability.^4^^,^^32^^–^^34^ These findings suggest that an inactive lifestyle and a poor nutritional status cause sarcopenia and subsequent ADL disabilities. Therefore, it may be reasonable to suppose that dietary intervention and the promotion of exercise habits would help to prevent sarcopenia and adverse health outcomes of geriatric disorders, such as ADL disability.

In a previously published systematic review, sarcopenia defined using the EWGSOP criteria was significantly associated with greater risks of mortality.^8^ Although all the studies included in this review followed the EWGSOP recommendation to use both low muscle mass and low muscle function for the diagnosis of sarcopenia, the measurement methods and the cut-off values for muscle mass and muscle function varied across the studies. The AWGS standardized the criteria of sarcopenia for Asian populations, which could improve comparability across studies. As far as we know, there have been only two population-based prospective longitudinal studies on sarcopenia risk in Asian populations, one conducted in Hong Kong and one in Japan, and both reported that sarcopenia determined using the AWGS definition was significantly associated with a higher risk of all-cause mortality.^4^^,^^9^ The present study also demonstrated that subjects with sarcopenia diagnosed using the AWGS definition had a significantly greater risk of mortality than those without. One possible explanation underlying the association between sarcopenia and mortality is that sarcopenia is linked with ADL disability and malnutrition,^8^^,^^35^ which have been shown to increase mortality risk.^36^^–^^38^ However, some residual confounding factors (eg, falls and increased susceptibility to infection) might exist because the association of sarcopenia with all-cause mortality remained significant after adjustment for ADL disability and nutritional status in this study.

In the present analysis, the magnitude in the excess mortality risk of sarcopenia weakened after additional adjustment for either handgrip strength or maximum gait speed. Notably, low handgrip strength and low gait speed, but not low muscle mass, were significant risk factors for all-cause mortality in the present study. These findings may suggest that handgrip strength and gait speed are risk factors for all-cause death in Japanese, without the need to consider skeletal muscle mass.

Several limitations of the current study should be noted. First, approximately 30% of study subjects were excluded from the analysis in both surveys, due to a lack of sufficient information for determining sarcopenia. The excluded subjects were more likely to be older and to have cognitive impairment and ADL disability than the included subjects in both surveys (eTable 6). Therefore, the prevalence of sarcopenia may have been underestimated in the current study. Second, the maximum gait speed was used for estimating sarcopenia in this study, instead of the usual gait speed. However, the correlation between usual gait speed and maximum gait speed appeared to be sufficiently strong (Pearson’s correlation coefficient: 0.76 for men, 0.82 for women). Moreover, we have examined the effects of usual versus maximum gait speed on the classification of sarcopenia by using the data from the 2017 survey. Of the 1,596 subjects, only 5 (0.3%) were misclassified due to the difference between usual gait speed and maximum gait speed. Therefore, the influence of this limitation should have been modest. Third, the causal relationships between sarcopenia and its associated factors cannot be inferred because of the cross-sectional design. Fourth, information on the number of hospital or institutional days before the baseline, which might have affected the presence of sarcopenia, was not available in the present study. Fifth, reverse causality might have occurred in the analysis of the mortality risk in subjects with sarcopenia. That is, sarcopenia may simply represent a poor physical condition before dying. However, the sensitivity analysis after excluding subjects who died during the first 2 years of the follow-up period did not alter the findings substantially (multivariable-adjusted HR 3.08; 95% CI, 1.53–6.19 for sarcopenia vs non-sarcopenia). Finally, the generalizability of the present findings was limited because participants were recruited from one town in Japan.

### Conclusions

The present study revealed that the prevalence of sarcopenia defined according to the AWGS criteria was approximately 7% in a community-dwelling older Japanese population. Subjects with older age and ADL disability were significantly more likely to have sarcopenia, whereas subjects with regular exercise and higher total energy intake were significantly less likely to have sarcopenia. Since these subjects with sarcopenia had greater mortality than those without, prevention of sarcopenia is needed to extend healthy life expectancy.

## APPENDIX A. SUPPLEMENTARY DATA

eTable 1. The detailed diagnostic patterns of sarcopenia in the enrolled and excluded groups at the survey in 2012 and in 2017

eTable 2. Crude and age-specific prevalence of sarcopenia in participants of the survey in 2017, when the usual gait speed was used for the estimation of sarcopenia

eTable 3. Clinical characteristics for subjects with and without sarcopenia at the survey in 2012

eTable 4. Risk of all-cause mortality in subjects with compared to those without sarcopenia or its components (adjusted for components of sarcopenia)

eTable 5. Influence of sarcopenia on all-cause death according to the subgroups of potential risk factors

eTable 6. Comparisons of clinical characteristics between the subjects included in and the subjects excluded from the analysis at the 2012 and 2017 surveys

eFigure 1. Flow diagram of the enrolled population at survey in 2012 (A) and at survey in 2017 (B).

eFigure 2. Sex-specific scatter plots of usual gait speed and maximum gait speed, the Hisayama Study, 2017. The black line indicates the linear regression.
