# Supplementary material for: Prevalence and Mortality of Sarcopenia in a Community-dwelling Older Japanese Population: The Hisayama Study
Source: J Epidemiol. 2021 May 5;31(5):320–7. doi: 10.2188/jea.JE20190289 (PMC8021883; doi:10.2188/jea.JE20190289)
Supplement: Supplementary file 1 [file je-31-320-s001.pdf]

eTable 1. The detailed diagnostic patterns of sarcopenia in the enrolled and excluded groups at the survey in 2012 and in 2017

	Low muscle mass	Low muscle function		Diagnosis of sarcopenia	Number of subjects	
		Low handgrip strength	Low gait speed		Survey in 2012	Survey in 2017
Enrolled in the study	Yes	Yes	Yes	Sarcopenia	18	13
			No		42	54
			Missing		29*	33*
		No	Yes		12	6
			No	Non-sarcopenia	195	223
		Missing	Yes	Sarcopenia	0	0
	No	Yes	Yes	Non-sarcopenia	19	22
			No		64	78
			Missing		29*	42*
		No	Yes		39	35
			No		782	916
			Missing		72*	95*
		Missing	Yes		0	0
			No		0	1*
			Missing		0	0
	Missing	No	No		70*	79*
	**Total number of enrolled subjects**				**1,371**	**1,597**
Excluded from the study	Yes	No	Missing	Unknown	16	21
		Missing	No		0	0
			Missing		0	1
	Missing	Yes	Yes		15	10
			No		20	13

			Missing		140	92
		No	Yes		9	4
			Missing		44	47
		Missing	Yes		0	0
			No		0	1
			Missing		247	314
	**Total number of excluded subjects**				**491**	**503**

* Sarcopenia status was determined using available data for the subjects lacking data for one or more diagnostic items for sarcopenia.

eTable 2. Crude and age-specific prevalence of sarcopenia in participants of the survey in 2017, when the usual gait speed was used for the estimation of sarcopenia

	Overall subjects		Men		Women	
	Subjects with sarcopenia[a] /total subjects	Prevalence (95% CI)	Subjects with sarcopenia[a] /total subjects	Prevalence (95% CI)	Subjects with sarcopenia[a] /total subjects	Prevalence (95% CI)
Crude prevalence, %	101/1,596[b]	6.3 (5.2–7.6)	37/705[b]	5.3 (3.7–7.2)	64/891	7.2 (5.6–9.1)
Age-specific prevalence, %						
65–69 years	6/507[b]	1.2 (0.4–2.6)	1/228[b]	0.4 (0.1–2.4)	5/279	1.8 (0.6–4.1)
70–74 years	13/414	3.1* (1.7–5.3)	5/188	2.7 (0.9–6.1)	8/226	3.5 (1.5–6.9)
75–79 years	14/321	4.4* (2.4–7.2)	4/131	3.1 (0.8–7.6)	10/190	5.3* (2.6–9.5)
80–84 years	34/223	15.3* (10.8–20.7)	14/106	13.2* (7.4–21.2)	20/117	17.1* (10.8–25.2)
≥85 years	34/131	26.0* (18.7–34.3)	13/52	25.0* (14.0–39.0)	21/79	26.6* (17.3–37.7)
p for trend		<0.001		<0.001		<0.001

CI, confidence interval.
Values are shown as the crude prevalence (95% CI).
[a] The usual gait speed was used for estimating sarcopenia.
[b] One subject was excluded, due to a lack of sufficient information for determining sarcopenia.
*$p<0.05$ vs. 65–69 years in each group (overall, men, and women).

eTable 3. Clinical characteristics for subjects with and without sarcopenia at the survey in 2012

Variables	Non-sarcopenia (n=1,270)	Sarcopenia [a] (n=101)	*p* value
Age, years	73.8 (6.2)	80.0 (7.3)	< 0.001
Sex, women, %	56.1	57.4	0.79
Height, cm	154.5 (8.9)	150.8 (8.4)	< 0.001
Weight, kg	56.1 (9.9)	46.1 (7.2)	< 0.001
BMI, kg/m^2	23.4 (3.2)	20.2 (2.5)	< 0.001
Obesity, %	28.0	3.0	< 0.001
Leanness, %	4.9	25.7	< 0.001
Living alone, %	9.9	10.9	0.75
Hypertension, %	70.8	71.3	0.91
Diabetes, %	24.9	21.8	0.49
Hypercholesterolemia, %	55.9	47.5	0.13
Serum albumin, g/dL	4.1 (0.2)	4.0 (0.3)	< 0.001
History of CVD or cancer, %	26.7	36.6	0.06
Cognitive impairment, %	18.7	37.6	< 0.001
History of fracture, %	36.4	40.6	0.40
ADL disability, %	4.6	20.8	< 0.001
Smoking habits, %	8.3	7.9	0.89
Alcohol intake, %	42.1	29.7	0.02
Regular exercise, %	19.8	7.9	0.004
Total energy intake, kcal/day	1,542 (341)	1,400 (270)	< 0.001
Energy balance, % of total energy intake			
Protein, %	12.4 (2.0)	12.0 (1.7)	0.04
Fat, %	25.3 (5.1)	25.2 (4.6)	0.73
Carbohydrate, %	56.9 (7.3)	58.5 (6.6)	0.03

ADL, activities of daily living; BMI, body mass index; CVD, cardiovascular disease.
Values are shown as the means (standard deviations) or frequencies.
[a] The maximum gait speed was used for estimating sarcopenia because the data on usual gait speed was unavailable in the 2012 survey.

eTable 4. Risk of all-cause mortality in subjects with compared to those without sarcopenia or its components (adjusted for components of sarcopenia)

	Model 1		Model 2		Model 3		Model 4	
	Hazard ratio (95% CI)	*p* value	Hazard ratio (95% CI)	*p* value	Hazard ratio (95% CI)	*p* value	Hazard ratio (95% CI)	*p* value
Sarcopenia [a]								
Absence	1.00 (reference)		1.00 (reference)		1.00 (reference)		1.00 (reference)	
Presence	2.20 (1.25–3.85)	0.006	3.24 (1.66–6.31)	<0.001	1.45 (0.75–2.78)	0.27	1.91 (1.02–3.58)	0.04
**Components of the definition for Sarcopenia**								
(Analyses with each component of the definition of sarcopenia as a categorical variable)								
Low muscle mass								
Absence	1.00 (reference)				1.00 (reference)		1.00 (reference)	
Presence	1.10 (0.67–1.80)	0.72			0.85 (0.50–1.43)	0.85	1.08 (0.63–1.84)	0.79
Low handgrip strength								
Absence	1.00 (reference)		1.00 (reference)				1.00 (reference)	
Presence	2.56 (1.55–4.24)	<0.001	2.94 (1.73–5.01)	<0.001			1.84 (1.10–3.45)	0.02
Low gait speed [a]								
Absence	1.00 (reference)		1.00 (reference)		1.00 (reference)			
Presence	2.02 (1.07–3.83)	0.03	2.08 (1.09–3.95)	0.03	1.60 (0.82–3.11)	0.17		
(Analyses with each component of the definition of sarcopenia as a continuous variable)								
Each 1SD decrement in SMI [b]	0.93 (0.70–1.22)	0.58			0.77 (0.58–1.03)	0.08	0.94 (0.70–1.28)	0.70
Each 1SD decrement in handgrip strength [b]	1.79 (1.28–2.52)	<0.001	2.04 (1.42–2.93)	<0.001			1.45 (0.96–2.17)	0.08
Each 1SD decrement in maximum gait speed [b]	1.64 (1.22–2.20)	0.001	1.64 (1.20–2.22)	0.002	1.47 (1.08–2.04)	0.02		

ADL, activities of daily living; CI, confidence interval; CVD, cardiovascular disease; SMI, skeletal muscle mass index; SD, standard deviation.
[a] The maximum gait speed was used for estimating sarcopenia and low gait speed because the data on usual gait speed were unavailable in the 2012 survey.
[b] The standard deviations of SMI, handgrip strength, and maximum gait speed were 1.1, 8.3, and 0.4, respectively.
Model 1 was adjusted for age, sex, hypertension, diabetes, history of CVD or cancer, history of fracture, ADL disability, smoking habits, alcohol intake, regular exercise, and total energy intake.
Model 2 was adjusted for the covariates included in model 1 plus SMI.
Model 3 was adjusted for the covariates included in model 1 plus handgrip strength.
Model 4 was adjusted for the covariates included in model 1 plus maximum gait speed.

eTable 5. Influence of sarcopenia on all-cause death according to the subgroups of potential risk factors

	Number of events/subjects	Hazard ratio (95% CI)	*p* for heterogeneity
Age <75 years	26/741	3.89 (1.19–12.69)	0.27
Age ≥75 years	57/574	2.47 (1.33–4.58)	
Women	31/736	1.67 (0.64–4.34)	0.47
Men	52/579	2.52 (1.23–5.16)	
BMI <22 kg/m^2	33/491	2.43 (1.08–5.46)	0.87
BMI ≥22 kg/m^2	50/824	2.27 (0.88–5.82)	
ADL disability (-)	73/1,241	2.19 (1.17–4.08)	0.90
ADL disability (+)	10/74	1.81 (0.25–13.31)	

ADL, activities of daily living; BMI, body mass index; CI, confidence interval.

Data were adjusted for age, sex, hypertension, diabetes, history of cardiovascular disease or cancer, history of fracture, ADL disability, smoking habits, alcohol intake, regular exercise, and total energy intake. The variable relevant to the subgroup was excluded from each model.

eTable 6. Comparisons of clinical characteristics between the subjects included in and the subjects excluded from the analysis at the 2012 and 2017 surveys

	Included subjects	Excluded subjects	*p* value between the included and excluded subjects
Survey in 2012			
Number of subjects	1,371	491	
Age, years	74.2 (6.5)	82.0 (8.1)	<0.001
Sex, women, %	56.2	67.2	<0.001
Cognitive impairment, %	20.1	51.0	<0.001
ADL disability, %	5.8	53.5	<0.001
Survey in 2017			
Number of subjects	1,597	503	
Age, years	74.1 (6.7)	82.0 (8.8)	<0.001
Sex, women, %	55.8	63.8	0.002
Cognitive impairment, %	17.8	51.1	<0.001
ADL disability, %	7.6	49.9	<0.001

ADL, activities of daily living.
Values are shown as the means (standard deviations) or frequencies.

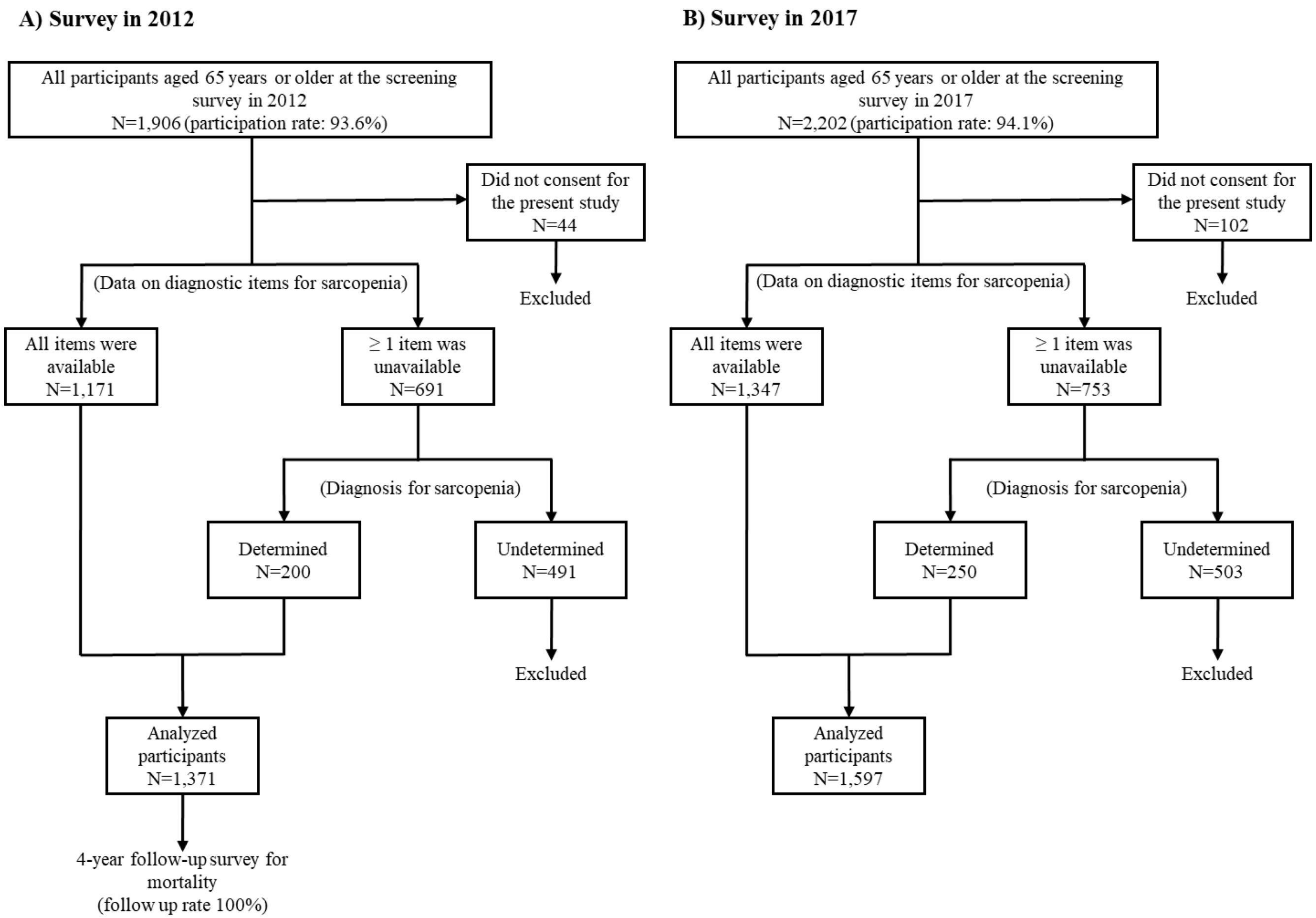

eFigure 1. Flow diagram of the enrolled population at survey in 2012 (A) and at survey in 2017 (B)

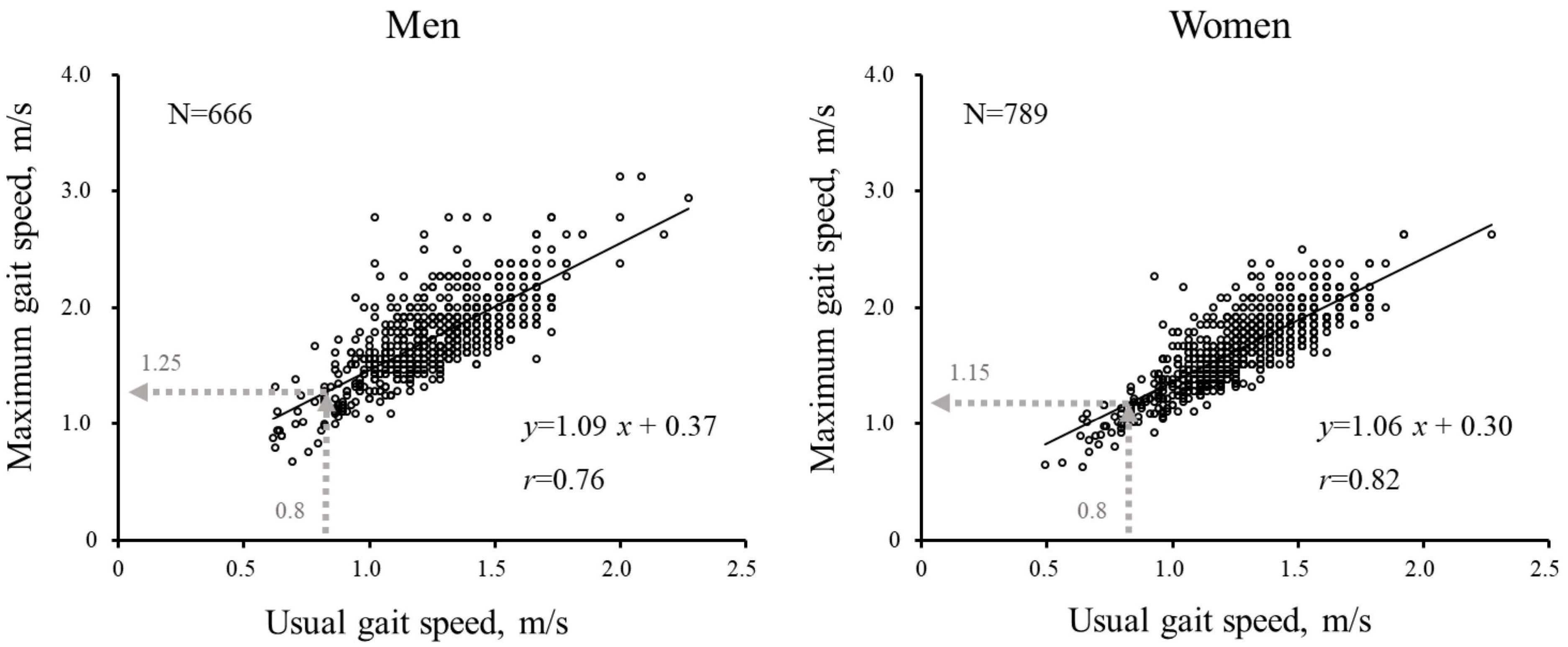

eFigure 2. Sex-specific scatter plots of usual gait speed and maximum gait speed, the Hisayama Study, 2017.
The black line indicates the linear regression.
